# From Alien Species to Alien Communities: Host- and Habitat-Associated Microbiomes in an Alien Amphibian

**DOI:** 10.1007/s00248-023-02227-5

**Published:** 2023-05-26

**Authors:** Franziska Leonhardt, Alexander Keller, Clara Arranz Aveces, Raffael Ernst

**Affiliations:** 1 Faculty of Biology, Technical University of Dresden, 01062 Dresden, Germany; 2 Museum of Zoology, Senckenberg Natural History Collections Dresden, Königsbrücker Landstraße 159, 01109 Dresden, Germany; 3 Faculty of Biology, Ludwig-Maximilians-University of Munich, Geschwister-Scholl-Platz 1, 80539 München, Germany; 4 Staatliches Museum für Naturkunde Stuttgart, Rosenstein 1, 70173 Stuttgart, Germany

**Keywords:** Amphibian microbiome, Skin microbiome, Gut microbiome, Environmental microbiota, Meta-communities, Microbial biogeography

## Abstract

Alien species can host diverse microbial communities. These associated microbiomes may be important in the invasion process and their analysis requires a holistic community-based approach. We analysed the skin and gut microbiome of *Eleutherodactylus johnstonei* from native range populations in St Lucia and exotic range populations in Guadeloupe, Colombia, and European greenhouses along with their respective environmental microbial reservoir through a 16S metabarcoding approach. We show that amphibian-associated and environmental microbial communities can be considered as meta-communities that interact in the assembly process. High proportions of bacteria can disperse between frogs and environment, while respective abundances are rather determined by niche effects driven by the microbial community source and spatial environmental properties. Environmental transmissions appeared to have higher relevance for skin than for gut microbiome composition and variation. We encourage further experimental studies to assess the implications of turnover in amphibian-associated microbial communities and potentially invasive microbiota in the context of invasion success and impacts. Within this novel framework of “nested invasions,” (meta-)community ecology thinking can complement and widen the traditional perspective on biological invasions.

**Supplementary Information:**

The online version contains supplementary material available at 10.1007/s00248-023-02227-5.

## Introduction

Invasive alien species (IAS) are a major component of the immense global environmental changes caused by human activities [1]. The number of alien taxa constantly increased over the last centuries [2], and they received considerable attention in both the scientific as well as the non-scientific communities [3–7]. Invasion biology research follows two main questions: (1) What are the impacts of alien species and how can they be translated in classifying invasive alien species [8–10]? And (2) what are the ecological and evolutionary drivers facilitating the establishment of alien species in novel environments [11–15]? This traditional perspective usually focuses on a single or several target taxa but largely neglects another potentially important component of invasions.

Alien species can host rather diverse microbial communities. These associated microbiota may play a substantial role in the invasion process. A well-studied example are rhizosphere microbiota that have been shown to contribute to plant fitness and the success of invasive alien plants [16]. While similar correlations have been reported for insects and their symbiotic microbiota [17], studies on alien vertebrates are scarce. This is particularly surprising for amphibians which maintain complex skin and gut microbiomes [18] but are also known to suffer from global population declines caused by the spread of microbial fungal pathogens [19]. This severe panzootic prompted numerous amphibian microbiome studies [20–23], mostly focussing on its contribution to amphibian health and changes caused by fungal infection. Yet, to the best of our knowledge, only three studies examined the microbiome of alien amphibians [24–26]). A systematic integration of microbiome data into (amphibian) invasion biology studies can complement our traditional perspective on invasive alien organisms and on what we here refer to as “nested micro-invasions”. This extended approach not only will help to understand the relevance of microbiota for invasion dynamics, but will also provide a unique framework to study microbiome assembly and variation.

The composition of the amphibian microbiome, in particular its intra- and inter-specific variation (see [18] for a review), is influenced by a combination of host-specific factors such as developmental stage and genotype [27, 28], and external factors such as microhabitat [29, 30] and climatic conditions [31]. The environmental microbial reservoir (microbial species pool) largely determines the composition of the skin microbiome [32, 33] and contributes to its intraspecific variation [30, 34]. This is consistent with the meta-community concept that considers microbial community assembly to be driven by both individualistic niche effects (i.e. host-specific and environmental factors) and exchange of microbes between microbial communities [35, 36]. Applying this concept to microbiomes of alien amphibians may enable us to quantify the exchange of microbiota with the environment and how this affects microbial community assembly in the gut and on the skin of the host organism. This is a first necessary step for further studies assessing the role of associated microbiomes for the invasion success of the host organism on one hand and the potential impact of translocated alien microbes on resident native ecosystems on the other hand.

Johnstone’s Whistling Frog, *Eleutherodactylus johnstonei*, is an ideal taxon for studying what can be regarded as nested invasions (i.e. associated microbiota being transferred by an alien host vector) in amphibians. The species is native to the Lesser Antilles and established alien populations on other Caribbean islands, the South and Central American mainland and in greenhouses in European botanical gardens [37, 38]. The broad spectrum of occupied microhabitats, spanning from natural forests in the native range (St Lucia) to urban habitats such as gardens (Colombia) and greenhouses (Europe) in the exotic range, provides a perfect setting for studying microbial community turnover along non-linear environmental gradients. A systematic assessment of the species’ associated microbiome also adds a second layer to the invasion biological studies on *E. johnstonei*, contributing a crucial aspect to the debate on its actual invasion potential [38–42].

Here we analysed the skin and gut microbiome of *E. johnstonei* from native range populations in St Lucia and exotic range populations in Colombia, Guadeloupe and several European greenhouse localities in combination with the respective environmental microbial reservoir. We hypothesize that amphibian-associated and environmental microbial communities form meta-communities that are determined by both selective niche effects and the transmission of microbes between communities. To quantify niche effects at different levels, we assess patterns of α- and β-diversities between microbial community sources (skin, gut, environment) and at different spatial scales (individuals, sites and regions). We expect (1) β-diversity of *E. johnstonei’*s associated microbiomes to increase with increasing distance and structural distinctness between native and exotic range environments. We assess proportions and abundance of shared microbial taxa between amphibian-associated and environmental communities to quantify interactions via a microbial transfer. We expect (2) interactions with the environmental microbiome to be most pronounced in frog associated microbial communities that are compositionally most differentiated from those in the native region.

## Methods

### Geographic Range and Sampling Sites

To represent the broad distribution and habitat range of *Eleutherodactylus johnstonei*, our dataset includes 16 populations from the native (St Lucia) and three exotic (Colombia, Guadeloupe, Europe) regions in different types of habitats (Table 1). Sampling area was comparable in all cases due to the spatial clustering and separation of identified populations.

**Table 1:** Sampled *E. johnstonei* populations. Geographic location of the sampling sites, habitat type and number of microbiome samples from skin, gut and environment (env) in the final (quality-filtered, see “Bioinformatic Processing”) dataset

	Region	Site	Site coordinates	Habitat type	skin/gut/env
Native	St Lucia (LCA)	Castries	14.014167, −60.979244	Urban (garden)	5/4/3
		Morne Le Blanc	13.761578, −60.997096	Natural (forest)	5/5/3
		Forest Ti Rocher	13.828985, −60.963323	Agricultural (plantation)	5/4/3
Exotic	Guadeloupe (GLP)	Grand-Bourg (Marie Galante)	15.883866, −61.317510	Urban (park)	4/5/3
		Le Gosier (Grande-Terre)	16.208928, −61.479177	Urban (fallow land)	5/5/3
		Sainte-Rose (Basse-Terre)	16.332981, −61.692132	Urban (garden)	4/2/3
Exotic	Colombia (COL)	Barranquilla	11.007486, −74.809155	Urban (park)	5/5/3
		Bucaramanga 1	7.118483, −73.106890	Urban (park)	4/5/3
		Bucaramanga 2	7.105774, −73.098301	Urban (fallow land)	5/5/3
		Cali	3.374177, −76.537406	Urban (park)	5/5/3
		Ibagué	4.445439, −75.205730	Urban (plant nursery)	4/6/3
Exotic	Europe (EUR)	Augsburg, DEU	10.913672, 48.349747	Greenhouse	3/3/2
		Frankfurt, DEU	8.657115, 50.124351	Greenhouse	2/3/2
		Halle, DEU	11.961172, 51.488806	Greenhouse	3/3/2
		Mainz, DEU	8.241462, 49.991097	Greenhouse	3/3/2
		Utrecht, NLD	5.171152, 52.090078	Greenhouse	3/3/1

### Microbiota Sampling

Soil samples (environmental microbiota), skin swabs and faeces (amphibian-associated microbiota) were collected from each site. Soil represents the most relevant environmental microbial community source for mainly terrestrial and partially fossorial (concealed in soil during low activity periods) host species. Each frog was captured with a sterilized falcon tube. Prior to skin swabbing each specimen was rinsed with 50 ml of sterile filtered and deionized (“PCR grade”) water to ensure sampling of skin-associated microbiota rather than debris and transient microbes (compare [24, 30, 34, 43, 44]). With a sterile cotton swab, each specimen was swabbed 10 times ventrally, 10 times dorsally, 5 times laterally on each side and 5 times on one hind leg including foot. After skin swabbing frogs were placed in a sterilized cricket-box with ~ 5 ml of sterile water (to avoid dehydration) overnight. Faecal pellets were collected the next morning with tweezers, sterilized by flaming with 70% ethanol after each sample. A fresh pair of sterile nitrile gloves was used for handling of each frog. All materials were sterilized by cleaning with 70% ethanol and subsequent UV-light irradiation for 30 min. At each population’s site soil samples were taken from the exact place where frogs were sampled with a sterile cotton swab. All samples (skin, gut and soil) were immediately placed in cryo-tubes with 99% ethanol which were stored at −20 °C after transportation to the laboratory. In accordance with official regulations in respective countries, frogs were either released at capture sites after handling (St Lucia, Colombia and Europe) or removed, euthanized and preserved as scientific vouchers deposited in museum collections (Guadeloupe; for a list of vouchers and details on collections, see [45]).

### Laboratory Workflow

DNA from skin, gut and environmental samples was isolated using the ZymoBIOMICS 96 DNA Kit (Zymo Research Europe GmbH, Freiburg, Germany) with the standard manufacturer’s protocol. The subsequent metabarcoding procedure of the 16S rRNA V4 region followed the dual-indexing strategy for library sequencing on Illumina MiSeq of Kozich et al. [46] (detailed protocol in Supplementary information S1).

### Bioinformatic Processing

Raw (Illumina) sequence data was processed using usearch v11 [47] and vsearch v2 [48] to obtain an amplicon sequence variant (ASV) table with counts of respective sequences per sample. Taxonomy was assigned (at 99% sequence identity) using the RDP 16S v16 reference database [49] and SINTAX for hierarchical classification (detailed protocol in Supplementary information S1). The dataset was further processed in R 4.0.5. [50] using the packages phyloseq [51], vegan [52] and edgeR [53]. Quality filtering was done by removing (1) all ASVs with an abundance of at least 5% in the negative controls (from DNA isolation and 16S amplification) to account for possible contamination; (2) taxa assigned as chloroplasts, mitochondria or only resolved to the domain level; and (3) samples with < 500 reads. As the resulting dataset covered a wide range of reads per sample (min = 661, max = 27226, mean = 8055; number of reads for each sample is given in Supplementary information S2), initial screenings were performed to ensure integrity with respect to α- and β-diversities of samples at the lower or upper end of the spectrum. For all analyses, except for calculations of α-diversity [54], the dataset was additionally low abundance filtered by excluding all ASVs with an abundance < 0.001% in the whole dataset [55] and normalized using the “TMM” method [56] of the edgeR extension for phyloseq following McMurdie and Holmes [54]. The final (quality filtered, low abundance filtered, normalized) ASV table, taxonomy table and sequences are provided in Supplementary information S2, S3 and S4.

### Community Analyses

All analyses were performed using R 4.0.5 if not stated otherwise. Alpha diversity at the ASV level was assessed as Shannon diversity using the phyloseq package [51]. ANOVA and Tukey tests were used to test for differences of α-diversity between microbial community sources (skin, gut, environment) and between the four regions (LCA, GLP, COL, EUR) for each source individually. The core microbiome, defined as genera with a prevalence of at least 90%, was identified for each community source across the whole range and for each region. Abundance of core genera was calculated as the average abundance in all samples of the respective subset. Abundance-based (quantitative) β-diversity of microbial communities was analysed using Bray-Curtis distances and visualized by non-metric multidimensional scaling (for individual samples). Permutational multivariate analysis of variance (PERMANOVA) [57, 58] was performed with the adonis function of the vegan package [52] to test for variation of microbial communities between sources, regions and sampling sites. PERMANOVA results were cross-validated using analysis of similarities (ANOSIM) [59, 60]. Differences in β-diversity among different sources and regions (for each source individually), i.e. community dispersal, were assessed with the multivariate homogeneity of groups dispersions approach of Anderson et al. [61]. The linear discriminant analysis (LDA) effect size (LEfSe) method [62] was used to identify differentially abundant taxa between a) the three microbiome sources (skin, gut, environment set as class variable, region set as subclass variable) and b) the four regions (LCA, GLP, COL, EUR set as class variable) for each source individually. LEfSe standard settings for the multi-class analysis strategy “one against all” were used. Regional differences of amphibian-associated microbiomes might be related to regional differences of environmental communities. Therefore, the overlap of taxa that are enriched in both the regional environment and regional skin/gut microbiomes, was identified. Proportions of shared and unique ASVs between the three community sources and between skin/gut and environmental microbiomes in each region, and at each site, were identified.

## Results

### Patterns of Microbial Community Diversity at Community Source and Spatial Scales

**Fig. 1:**
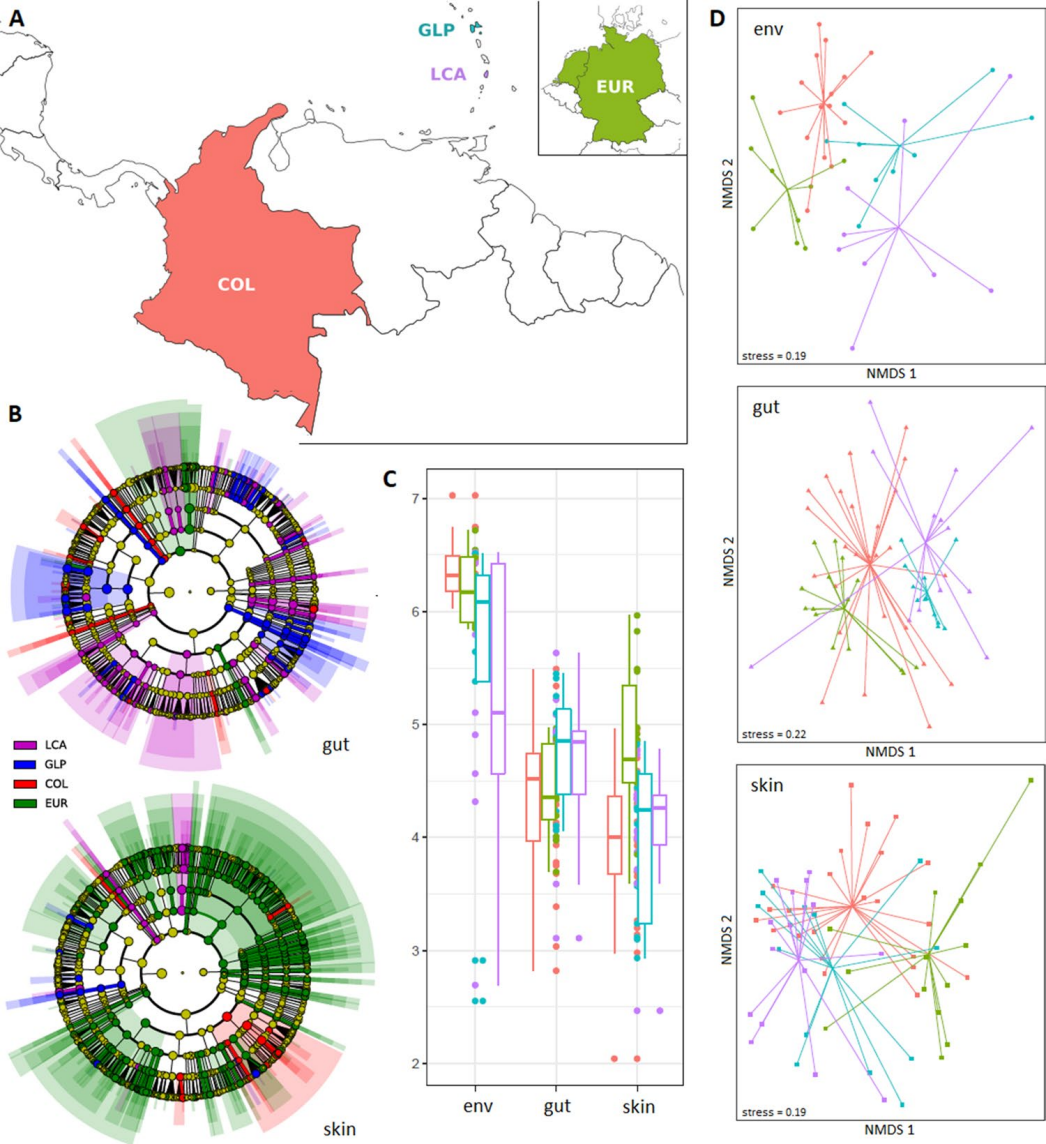
Geographic (regional) patterns in associated microbiomes of *E. johnstonei* and environmental microbiomes. (**A**) Microbiomes were investigated in the native region St Lucia (LCA, purple) and the exotic regions Guadeloupe (GLP, blue), Colombia (COL, red) and Europe (EUR, green; greenhouse populations in Germany and The Netherlands); regional colour codes correspond to colours in (**B**), (**C**) and (**D**). (**B**) Regionally enriched taxa (coloured by region) in gut and skin microbial communities; 18% of all taxa (on all phylogenetic levels) are regionally enriched in gut microbiomes and 27% in skin microbiomes. (**C**) Shannon (alpha) diversity of environmental, gut and skin samples from the four regions. (**D**) Non-metric multidimensional scaling of individual environmental, gut and skin samples based on Bray-Curtis dissimilarity with group centroids for each region

Alpha diversity (Shannon diversity, α_H_) was significantly different between skin, gut and environmental microbial communities (ANOVA, *Df* = 2, *F* = 65.4, *p* = 8.73e−22, *p*_BHadj_ = 3.49e−21; Fig. 1C; Table 2). Significant regional differences of *α-*diversity were detected for skin and environmental microbiomes (ANOVA, skin: *Df* = 3, *F* = 5.6, *p* = 0.002, *p*_BHadj_ = 0.004, env: *Df* = 3, *F* = 4.5, *p* = 0.009, *p*_BHadj_ = 0.01), but not for gut microbiomes (ANOVA, *Df* = 3, *F* = 1.9, *p* = 0.14, *p*_BHadj_ = 0.14). Pairwise comparisons (TUKEY test) revealed that skin microbiomes of *Eleutherodactylus johnstonei* in Europe were more diverse than those of the other regions (*p*_adj_(EUR-COL) = 0.002; *p*_adj_(EUR-GLP) = 0.01; *p*_adj_(EUR-LCA) = 0.03; Table 2). Environmental microbiomes were most diverse in Colombia and least diverse in St Lucia (TUKEY, *p*_adj_(COL-LCA) = 0.02; Table 2). All other pairwise comparisons did not reveal significant regional differences of α-diversity.

**Table 2:** Composition and diversity of microbial communities at community source and regional scales. Each parameter is given for each community source (skin, gut, environment) across the whole sampled range of *E. johnstonei* (first line) and in individual regions (second line)

	Skin				Gut				Environment			
	LCA	GLP	COL	EUR	LCA	GLP	COL	EUR	LCA	GLP	COL	EUR
Shannon diversity, $\overline{\alpha}$_H_	4.2				4.5				5.9			
	4.1	4.0	3.9	4.8	4.6	4.8	4.3	4.5	5.2	5.3	6.4	6.2
Core microbiome (no. genera, abundance)	4, 0.35				27, 0.80				42, 0.58			
	11, 0.65	5, 0.42	3, 0.31	19, 0.42	22, 0.71	84, 0.97	29, 0.82	30, 0.88				
No. differentially abundant taxa	12				30				157			
	16	14	18	245	73	71	26	19	33	33	199	85
Community dispersion, d	0.60				0.57				0.61			
	0.54	0.58	0.57	0.58	0.57	0.36	0.56	0.51	0.57	0.56	0.52	0.57

Overall, we detected 6174 ASVs (in 644 genera, 228 families, 110 orders, 60 classes, 25 phyla) in the whole dataset after low abundance filtering and normalization. Out of these, 3741 ASVs were present in skin, 4147 in gut and 5156 in environmental microbial communities. The skin core microbiome (genera with a prevalence ≥ 90% of all samples) across the whole studied range of *E. johnstonei* consisted of four genera only (*Acinetobacter, Pseudomonas, Bacteroides, Comamonas*) and had an average abundance of 35% in all skin samples. In regional skin samples, the core microbiome made up the highest proportion (average abundance in all skin samples of the respective region) in St Lucia, while it was less abundant in all three exotic regions (Table 2). In comparison to skin, the gut core microbiome across the whole range consisted of more genera (27) and made up a higher proportion (80%) of the gut microbiome. In regional gut samples, the core microbiome made up the lowest proportion in St Lucia, while its abundance was higher in all three exotic regions (Table 2). The environmental core microbiome consisted of even more genera (42) and made up 58% of the environmental microbiome. All (regional) core genera of skin, gut and environmental microbial communities are listed in Supplementary information S5.

A total of 209 taxa (across all taxonomic levels, from genera to phyla) showed differential abundance between the three community sources, with most taxa enriched in environmental communities (Table 2, taxa are listed in Supplementary information S6). We further tested for regional differential abundance of taxa in skin, gut and environmental microbial communities individually (see Supplementary information S6). Skin microbiomes of *E. johnstonei* in Europe showed the highest number of enriched taxa, while these numbers were substantially lower in the remaining regions (Fig. 1B; Table 2). In gut microbiomes, more taxa were enriched in the two Caribbean regions than in the non-Caribbean regions (Fig. 1B; Table 2). In environmental microbial communities, 350 taxa (33%) showed regional differential abundance; mainly in Colombia and less in the remaining regions (Table 2).

**Fig. 2:**
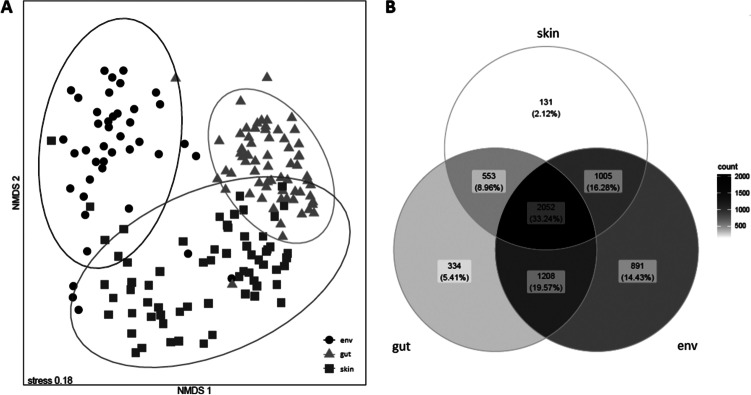
Community composition (abundance-based β-diversity) and shared taxa for microbial communities by source (environmental, skin and gut). **A** Non-metric multidimensional scaling of individual samples based on Bray-Curtis dissimilarity, with 0.95 ellipsoids assuming multivariate t-distributions for each source. **B** Venn diagram of pooled skin, gut and environmental samples at the ASV level; colour gradation from white to black indicates increasing counts of ASVs

Abundance-based microbial community composition (measured by Bray-Curtis dissimilarity) was partly explained by community source (Fig. 2A), as well as by the spatial parameter region (Fig. 1D) and site of respective *E. johnstonei* populations. Within the whole data set, source was the single parameter explaining the highest proportion of variance (15%), followed by site (8%) and region (4%) (PERMANOVA; source: *Df* = 2, *F* = 18.6, *R*^2^ = 0.15, *p* = 0.001; region: *Df* = 3, *F* = 3.6, *R*^2^ = 0.04, *p* = 0.001; site: *Df* = 12, *F* = 1.8, *R*^2^ = 0.08, *p* = 0.001). With 16%, interactions between source and site explained most variation and interactions between source and region explained 9% of variation (source × site: *Df* = 24, *F* = 1.7, *R*^2^ = 0.16, *p* = 0.001; source × region: *Df* = 6, *F* = 3.8, *R*^2^ = 0.09, *p* = 0.001). In total, the three parameters and their interactions explained 51% of variance (*Df* = 47, *F* = 2.8, *R*^2^ = 0.51, *p* = 0.001). When considering the three sources individually, region and site explained 36% of variation in skin (region: *Df* = 3, *F* = 2.9, *R*^2^ = 0.12, *p* = 0.001; site: *Df* = 12, *F* = 1.5, *R*^2^ = 0.24, *p* = 0.001), 46% of variation in gut (region: *Df* = 3, *F* = 5.5, *R*^2^ = 0.18, *p* = 0.001; site: *Df* = 12, *F* = 2.2, *R*^2^ = 0.28, *p* = 0.001) and 51% of variation in environmental microbial communities (region: *Df* = 3, *F* = 3.1, *R*^2^ = 0.18, *p* = 0.001; site: *Df* = 12, *F* = 1.5, *R*^2^ = 0.33, *p* = 0.001). The results of ANOSIM cross-validated the PERMANOVA results by confirming similarity of the community composition within sources, regions and sites (ANOSIM; source: *R* = 0.65, *p* = 0.001, *p*_BHadj_ = 0.002; region: *R* = 0.04, *p* = 0.014, *p*_BHadj_ = 0.014; site: *R* = 0.05, *p* = 0.005, *p*_BHadj_ = 0.006) and especially within sources at the same sites (source × site: *R* = 0.70, *p* = 0.001, *p*_BHadj_ = 0.002) and sources in the same regions (source × region: *R* = 0.63, *p* = 0.001, *p*_BHadj_ = 0.002).

Community dispersion (i.e. within group variation of community composition) was significantly different between microbial communities of the different sources (ANOVA, *Df* = 2, *F* = 4.5, *p* = 0.01; Table 2). It was lower in gut communities as compared to skin (Tukey HSD: *p*(gut-skin) = 0.03) and environmental communities (Tukey HSD: *p*(gut-env) = 0.03), while there was no significant difference between skin and environmental communities (Tukey HSD: *p*(skin-env) = 0.98). Regional variation of community dispersion was only detected for gut microbial communities (ANOVA(skin): *Df* = 3, *F* = 0.9, *p* = 0.46; ANOVA(env): *Df* = 3, *F* = 1.2, *p* = 0.34; ANOVA(gut): *Df* = 3, *F* = 16.1, *p* = 7.34e−08), which showed significantly lower dispersion in Guadeloupe as pairwise compared to the other three regions (Tukey HSD: *p*(LCA-GLP) = 0.1e−05, *p*(COL-GLP) = 0.1e−06, *p*(EUR-GLP) = 0.0002; Fig. 1D; Table 2).

Inter-region (Bray-Curtis) dissimilarity between skin microbial communities of exotic and native *E. johnstonei* samples was significantly different in the three exotic regions (Kruskal-Wallis: *p* < 2.2e−16; mean pairwise Bray-Curtis dissimilarities: LCA-GLP = 0.816, LCA-COL = 0.834, LCA-EUR = 0.918). A post hoc Dunn’s test revealed that European skin communities were significantly more distinct from St Lucian skin communities than those in Guadeloupe (*p*_Bonf_ = 0.00) and Colombia (*p*_Bonf_ = 0.00). Also gut microbial communities in the three exotic regions show significant differences in their dissimilarity to the native gut communities (Kruskal-Wallis: *p* < 2.2e−16; mean pairwise Bray-Curtis dissimilarities: LCA-GLP = 0.764, LCA-COL = 0.866, LCA-EUR = 0.879). Gut communities in Guadeloupe were significantly less dissimilar from native gut communities than those in Colombia (*p*_Bonf_ = 0.00) and Europe (*p*_Bonf_ = 0.00).

### Interactions Between Amphibian-Associated and Environmental Microbial Communities

The Venn diagram of ASVs pooled for all samples of each source (Fig. 2B) shows that most ASVs are shared between all three sources (33.24%) and that the proportions of ASVs shared between each amphibian-associated source and the environment (skin × env: 16.28%, gut × env: 19.57%) were considerably higher than those shared between skin and gut (8.96%). Only small proportions of ASVs occurred exclusively in skin (2.12%) and gut (5.41%), while environmental communities contained a higher proportion of unique ASVs (14.43%).

**Fig. 3:**
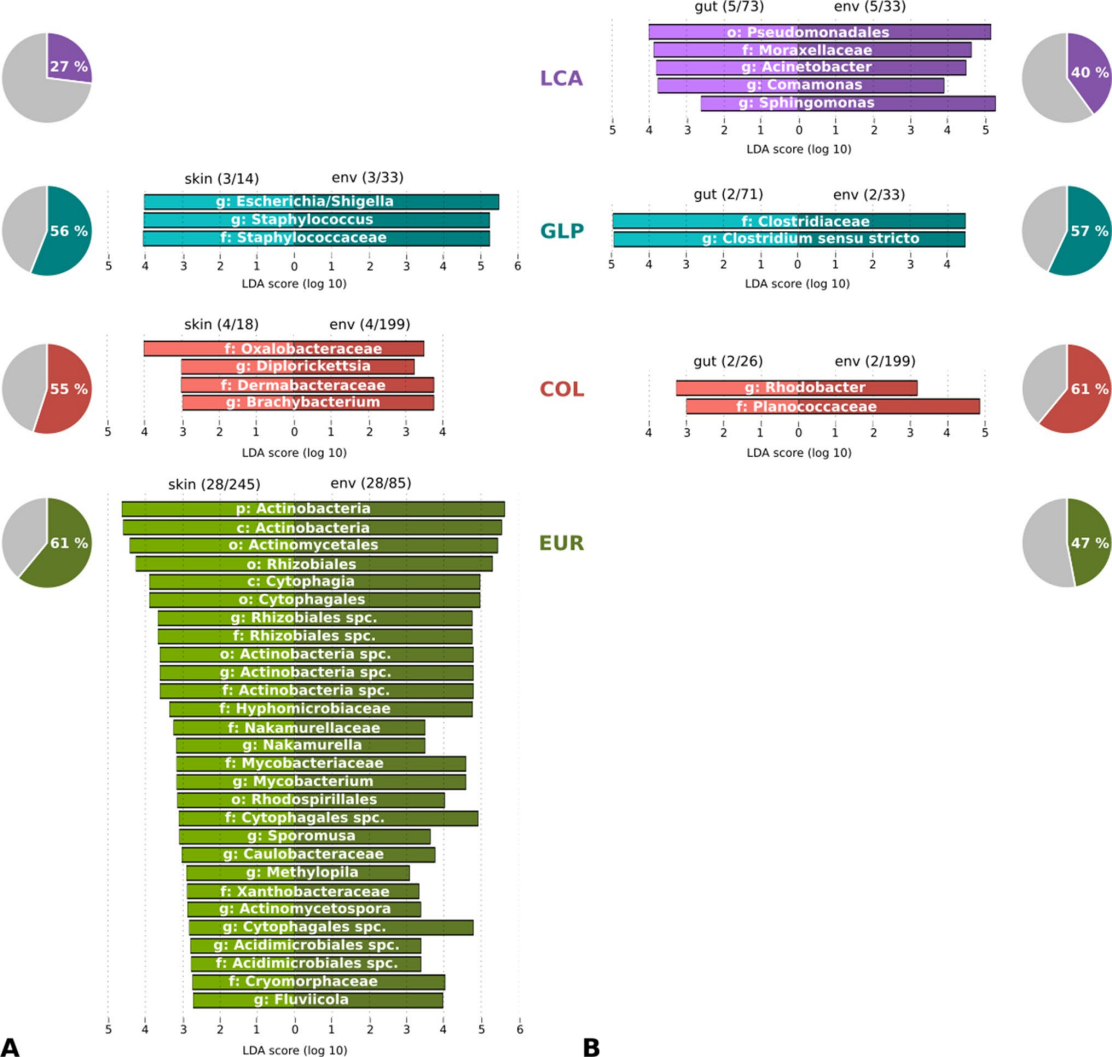
Interactions between *E. johnstonei*’s associated (**A** skin, **B** gut) and environmental microbial communities in the native region St Lucia (LCA, purple) and the three exotic regions Guadeloupe (GLP, blue), Colombia (COL, red) and Europe (EUR, green). For amphibian-associated and environmental communities of each region, shared microbes on the species/ASV level (pie charts) and interdependencies between regional compositional differences (bar charts) are illustrated. Pie charts show the proportions of skin (**A**) or gut (**B**) ASVs, pooled per region, overlapping with environmental ASVs of the same region. Bar charts show LDA scores for taxa that are enriched in both regional skin (**A**) or gut (**B**) microbiomes and regional environmental (env) microbiomes. The proportions in brackets indicate how many taxa are enriched in total in the respective skin/gut/env microbiomes of the region, the phylogenetic levels of enriched taxa are given as genus (g), family (f), order (o), class (c) and phylum (p)

We further identified shared ASVs between amphibian-associated and environmental microbiomes on the site and region level (see pie charts in Fig. 3, A: skin, B: gut). In all exotic regions of *E. johnstonei*, more skin ASVs were shared with the environment of the same region (GLP: 56%, COL: 55%, EUR: 61%) than in the native region (LCA: 27%). This was also confirmed on the site level, where on average 15.7% of the skin ASVs were shared with the environment at sampling sites in St Lucia, while this proportion was significantly higher at sites in the exotic regions (GLP: 31.2%, COL: 33.1%, EUR: 30.3%, *p*(*t*) = 0.0004). Also gut microbiomes (Fig. 3B) share more ASVs with the environment in the exotic regions as compared to the native region, which is true on both regional (LCA: 40%, GLP: 57%, COL: 61%, EUR: 47%) and site level (LCA: 18%, GLP: 30.7%, COL: 35.8%, EUR: 19.8%, *p*(*t*)_LCA<exotic_ = 0,02).

To test for interdependencies between regional differences in amphibian-associated microbiomes and regional differences in environmental communities, we identified overlap of enriched taxa in both environmental and skin/gut microbiomes of each region (see bar charts in Fig. 3, A: skin, B: gut). Regional differences in environmental communities were strongly reflected in skin communities of *E. johnstonei* in Europe. Twenty-eight taxa were enriched in both European environment and skin microbiomes, while the same applies for only 4 taxa in Colombia, 3 taxa in Guadeloupe and none in St Lucia. Gut communities showed an opposite pattern. Most regionally enriched taxa in gut communities were overlapping with taxa enriched in the respective regional environmental communities in St Lucia, followed by Guadeloupe and Colombia (LCA: 5/73, GLP: 2/71, COL: 2/26), while none of the enriched taxa in gut communities of *E. johnstonei* in Europe was enriched in environmental samples in Europe.

## Discussion

Concepts of community ecology are at the heart of a holistic understanding of the dynamics and mechanisms of biological invasions [63, 64]. This is particularly true when assessing complex and diverse microbiota associated with the target taxa in classical invasion studies. Traditional invasion studies have largely failed to integrate this nested invasion component and therefore ignored a potentially important aspect of invasion processes. In our study we show that microbial communities associated with introduced alien amphibians and environmental microbial communities can be considered as meta-communities that interact and exchange components in an ecological assembly process. Just as in other ecological communities, compositional patterns are determined by a mixture of selective ecological filters, spatial configuration of species pools and mere chance events [36, 65, 66]. The observed differences in microbial community composition within and between native (St Lucia) and exotic (Guadeloupe, Colombia, Europe) populations of *Eleutherodactylus johnstonei* indicate a potential transfer of microbiota between frogs and environment. While actual effects on the invasion success and impacts on native biota, including native microbial assemblages remain speculative and require extended experimental approaches, our results highlight the relevance of investigating nested invasions of alien hosts more systematically. Identifying and analysing these patterns is a crucial step forward in understanding the relevance of environmental transfer and spatial factors on variability and biogeography of the (amphibian) microbiome.

Abundance-based (quantitative) β-diversity of the microbial communities in our study was primarily determined by the source supporting these communities (skin, gut or environment). Scaled spatial factors (region vs. site) explained only small proportions of the community dissimilarity. This observed community source dependence mirrors previously reported compositional patterns in amphibian communities at macro-ecological scales (compare [67]). General community source properties therefore seem to have a stronger (deterministic) effect on microbial community composition than spatial factors. Other studies equally report marked differences between amphibian-associated and environmental microbial communities [30, 33, 34, 68]. However, when considering presence/absence based β-diversity only, we found differences to be less pronounced. In line with previous studies [30, 68] we found that large proportions of ASVs were present in both skin and environmental (80%) or gut and environmental (79%) communities. This suggests that microbes disperse between environmental and amphibian-associated communities, while respective abundances are rather determined by niche effects that are mainly driven by source (skin, gut) properties. This is consistent with the meta-community concept of species sorting [35] and has previously been reported for microbial communities in different environments, ranging from freshwater to animal tissues [69].

While microbial community sources largely determine compositional patterns, variation within skin and gut microbiomes of *E. johnstonei* was still considerably high. Spatial patterns of β-diversity were highly nested, with sites representing regional subsets in both skin and gut communities. Thus, environmental filters seem to primarily affect the microbiome at the site level. Yet, microbiomes also differed by region as similarly reported for skin microbiomes of the cane toad, *Rhinella marina* [24] and gut microbiomes of the guttural toad, *Sclerophrys gutturalis* [26]. We observed increasing difference from native region microbiomes relative to the geographic distance (increasing from GLP to COL to EUR). Two external environmental factors likely contribute to this observed pattern. (1) Habitats of more distant exotic populations of *E. johnstonei* show greater structural differences from native habitats, i.e. mostly urban, but also present in agricultural and natural habitats in GLP [39, 70] (pers. obs.), exclusively urban in COL [38] and fully artificial (greenhouses of botanical gardens) in EUR (pers. obs.). (2) Macroclimatic conditions change dramatically with increasing distance from the native source region. We also detected considerable individual variation of skin and gut microbiomes that remains unexplained by spatial factors. A recent study shows that habitat disturbance can result in greater microbial dispersion of the amphibian skin microbiome [71], which provides a possible explanation for the observed individual variation in our study. However, stochastic assembly processes likely play a major role in microbiomes [33, 72] and therefore strongly influence individual composition patterns.

While skin microbiomes in our study exhibited great spatial variation (compare [28, 68, 73]), presumably due to their higher sensitivity to external environmental conditions [23, 31, 74–76], gut microbiomes of *E. johnstonei* appeared less variable (lower β-diversity) but at the same time showed high levels of α-diversity. Previous studies found gut microbiomes to be strongly determined by diet [75, 77], which is diverse in exotic populations of *E. johnstonei* in disturbed urban habitats [78–80]. This diverse diet probably contributes to high alpha diversity of gut microbiomes across the whole range while preventing strong differentiation (β-diversity).

We suggest that two main mechanisms cause the geographic patterns detected in *E. johnstonei’s* associated microbiomes. While external, abiotic factors can modify local abundances of present microbial taxa, transmissions from the environmental microbial reservoir are the source of new taxa (compare [36, 66]). In combination with vertical (from parents to offspring) and horizontal (intra- and inter-specific) transmissions between individuals, these processes are responsible for the establishment and maintenance of the amphibian microbiome, while knowledge on their relative contribution is still largely lacking [81, 82]. The few studies on transfer of microbes between amphibian life stages found that the bacterial communities in clutches initially resemble those found in adults [82, 83] and microbial assemblages of tadpoles later converge to a certain degree towards those found in their aquatic environment [82]. Studies on (environmental, horizontal, or vertical) microbial transfer in direct developing species with extensive parental care, such as *E. johnstonei* [84] are missing so far. The accumulated evidence indicates a mixed-mode transmission [85] that maintains the amphibian microbiome, whereby environmental transmissions seem to have a higher relevance for the skin than the gut microbiome of *E. johnstonei* and thus contribute to the observed geographic patterns.

For both skin and gut microbiomes, we found similarly high proportions of taxa possibly bi-directionally transferred from/into environmental communities, while regionally higher intensities of such interactions were associated with stronger compositional changes only in skin microbiomes. The bacterial phyla Thaumarchaeota, Acidobacteria and Planctomycetes, for example, represent the dominant taxa in soils world-wide [86, 87], and they were highly enriched in skin samples of European greenhouse populations but not in gut samples. Likewise, reciprocal transfer experiments of European fire salamander larvae (*Salamandra salamandra*) show that gut microbiomes shift differentially resulting in habitat specific functional composition as opposed to more direct taxonomic turnover of skin microbiomes [75].

The detected microbiome turnover in alien populations of *E. johnstonei* could have several functional implications. Changes of the microbiome are magnitudes faster than other (genetic or epigenetic) adaptation mechanisms and therefore are likely important when rapid responses to new environmental conditions are required [88]. Studies on the human intestinal microbiome suggest that alterations can either act as a fast-response mechanism to adjust metabolic functions to dietary or environmental changes [89] or might even induce long-term evolutionary mechanisms for climate change adaptation [90]. However, changes of the microbiome might also pose negative effects, like microbiome dysbiosis. This imbalance of the microbial community negatively affecting its functionality can not only be caused by pesticides or other chemicals but also by habitat degradation/disturbance [18, 71, 91, 92]. To test whether the observed microbiome variation implies negative, positive or neutral effects on *E. johnstonei,* experimental setups, such as fitness tests and/or transfer experiments would be required. These experiments could also elucidate temporal patterns of microbiome variation and should be systematically explored in future studies.

Our results indicate intensified interactions between environmental and amphibian-associated microbiomes in the exotic regions, which imply a bi-directional transfer of microbiota. While we have direct evidence for the transfer of microbiota from the environment to the frogs, based on comparison between native and exotic microbiomes, the transfer from frogs to the environment remains hypothetical at this stage. Elucidating this aspect would require intensive microbial community sampling across large spatial scales to generate a reference data base, which is currently lacking. Shared occurrences of microbiota (between frogs and environment) can result from two possible scenarios: (1) Vertical transfer of microbiota from parents to offspring over generations and subsequent “invasion” of environmental communities in each new generation and (2) spillover of microbiota to the environment immediately after introduction of the first individuals with subsequent establishment of these microbiota in the novel environment and spillback. In this way, the environment would act as a transmission hub for microbial exchange within and between species [93]. Such microbial transfer processes should ideally be assessed by experimental approaches, e.g. a comparison of amphibian-associated and environmental microbiomes before and after transferring frogs to artificial or “semi-sterile environments” [94]. For amphibians, direct and indirect (through the environment) horizontal transmission was proven for antifungal bacteria [95], as well as the amphibian pathogen *Batrachochytrium dendrobatidis* [21, 96]. Dispersal of microbiota by alien species should receive more attention, as not only vector-borne pathogens but also non-pathogenic microbes can become invasive [97–99]. The impacts are multifaceted including the disturbance of biochemical cycles or evolutionary effects on native microbial communities [97, 100].

The novel framework of “nested invasions”, which we here introduce for the first time, heavily relies on (meta-)community ecology thinking in an invasion biology context. It provides an avenue for a more holistic approach to the analysis of alien species as it considers the alien host and its associated microbiome as an interacting entity that should be assessed together. Alien species do not come alone, and the broader application of the meta-organism concept [101] can complement and widen the traditional perspective on biological invasions.

### Supplementary Information

ESM 1: **Supplementary information S1** Detailed protocols of the laboratory workflow and bioinformatic processing. (PDF 730 kb)

ESM 2: **Supplementary information S2** ASV table of the final (quality filtered, low abundance filtered, normalized) dataset. For each sample, the number of reads for each ASV are given; sample names include sampling site and community source (skin, gut, or environment [env]). (XLSX 3342 kb)

ESM 3: **Supplementary information S3** Taxonomy table including phylum (p), class (c), order (o), family (f), and genus (g) for each ASV. (XLSX 253 kb)

ESM 4: **Supplementary information S4** Sequences for each ASV. (XLSX 490 kb)

ESM 5: **Supplementary information S5** Environmental, gut, and skin core microbiomes across the whole sampled range, and for each region individually. For each region (whole range, LCA, GLP, COL, EUR), all core genera, their prevalence and abundance (average abundance in all samples of the respective subset) are given. (XLSX 23 kb)

ESM 6: **Supplementary information S6** Differentially abundant microbial taxa identified with LefSe between the three sources (skin, gut, environment) and between the four regions (LCA, GLP, COL, EUR) for each source individually. For each differentially abundant taxa, the class that is characterized by its differential abundance (skin/gut/env or LCA/GLP/COL/EUR for the individual sources), LDA effect size, and p-values are given. (XLSX 57 kb)

## Data Availability

Raw sequence data is available in the NCBI Sequence Read Archive under the BioProject PRJNA911332 (https://www.ncbi.nlm.nih.gov/sra/PRJNA911332). ASV table and ASV sequences are provided in Supplementary information S2 and S4.
