# Supplementary material for: From Alien Species to Alien Communities: Host- and Habitat-Associated Microbiomes in an Alien Amphibian
Source: Microb Ecol. 2023 May 26;86(4):2373–85. doi: 10.1007/s00248-023-02227-5 (PMC10640505; doi:10.1007/s00248-023-02227-5)
Supplement: Supplementary file 1 — Supplementary information S1 Detailed protocols of the laboratory workflow and bioinformatic processing. (PDF 730 kb) [file 248_2023_2227_MOESM1_ESM.pdf]

Supplementary information S1

Laboratory workflow

DNA isolation

Genomic DNA from skin, gut, and environmental samples was isolated using the ZymoBIOMICS 96 DNA Kit (Zymo Research Europe GmbH, Freiburg, Germany) with the standard manufacturer's protocol and ZR Bashing Bead Lysis Tubes (in a vertical adapter for the Vortex Genie).

The subsequent metabarcoding of the 16S rRNA V4 region followed the dual-indexing strategy for library sequencing on Illumina MiSeq of Kozich et al. (2013), a more detailed protocol can be found in their supplementary information; any deviations are covered in following outlined overview of the procedure.

PCR

- The 16S V4 variable region was amplified using a unique pair of indexed primers (Illumina 2021) for each sample
    - AATGATACGGCGACCACCGAGATCTACAC [8bp-i5 index] ATGGTAATTGTGTGCCAGCMGCCGCGGTAA (forward)
    - CAAGCAGAAGACGGCATACGAGAT [8bp-i7 index] AGTCAGTCAGCCGGACTACHVGGGTWTCTAAT (reverse)
- To reduce random effects PCR reactions were performed in triplicates for each sample; for 3 reactions of 10 µl each, 30 µl master mix were prepared containing:
    - 0.6 Units Phusion High Fidelity DNA-Polymerase (Thermo Fisher Scientific, Waltham, MA, USA)
    - 0.2 mM dNTPs)
    - 1x Phusion HF Buffer (6 µl)
    - 1 µl of each forward/reverse 10mM primer
    - up to 5 µl of template DNA
- PCR conditions: denaturation at 95 °C for 4 min, amplification using 37 cycles of 95 °C for 45 sec, 49 °C for 40 sec and 72 °C for 40 sec, and final extension at 72 °C for 5 min
- Triplicates were combined after PCR and success was checked through gel electrophoresis using a 1% agarose gel.

Negative controls using PCR grade dH2O for DNA isolation (DNAneg) and for PCR (PCRneg) were processed in the same laboratory procedure.

Normalization and pooling

- The DNA amount was normalized between samples using, the SequalPrep™ Normalization Plate (96 well) Kit (Invitrogen, Thermo Fisher Scientific, Carlsbad, CA, USA).
- Fragment length distributions were verified using d1000 assays for the 4200 TapeStation (Agilent Technologies, Santa Clara, CA, USA).
- Pooling strategy:
    - Three pools were created according to amplicon quantity of individual samples
    - 4200 TapeStation and Qubit 3.0 Fluorometer (Invitrogen, Thermo Fisher Scientific, Carlsbad, CA, USA) were used for quality control and quantification

- Pools were cleaned-up and concentrated with the MinElute PCR Purification Kit (Qiagen, Venlo, Netherlands)
- The 3 pools were combined in equal molarities to the final pooled 16S amplicon library

- The final pool was quantified using the Qubit 3.0 Fluorometer.

Sequencing

Sequencing was performed on a Illumina MiSeq with a v2 2x 250bp chip according to the protocol of Kozich et al. (2013) and the manufacturer's description.

Bioinformatic processing (details on usearch/vsearch processing)

- Forward and reverse reads were merged with fastq_mergepairs (minimum merge length 100 bp, maximum differences 10 bp).
- Quality was ensured by truncating merged and unmerged sequences to a maximum expected error of 1.5 and filtering out sequences < 200 bp.
- To determine ASVs (amplicon sequence variants), sequences were dereplicated, singletons removed, denoised and chimeras removed.
- ASVs were mapped to sample reads by 97 % sequence identity for counts per sample.

References

Illumina (2021) Illumina Adapter Sequences. Available from: https://support-docs.illumina.com/SHARE/AdapterSeq/illumina-adapter-sequences.pdf.

Kozich JJ, Westcott SL, Baxter NT, Highlander SK, Schloss PD (2013) Development of a Dual-Index Sequencing Strategy and Curation Pipeline for Analyzing Amplicon Sequence Data on the MiSeq Illumina Sequencing Platform. Applied and Environmental Microbiology 79: 5112–5120. https://doi.org/10.1128/AEM.01043-13
